# Negotiating the caring role and carer identity over time: ‘living well’ and the longitudinal narratives of family members of people with dementia from the IDEAL cohort

**DOI:** 10.1017/S0144686X25000030

**Published:** 2025-03-18

**Authors:** Sally Stapley, Claire Pentecost, Catherine Quinn, Christina Victor, Jeanette Thom, Catherine Henderson, Isla Rippon, Serena Sabatini, Linda Clare

**Affiliations:** 1 REACH: The Centre for Research in Ageing and Cognitive Health, University of Exeter Medical School, Exeter, UK; 2 Centre for Applied Dementia Studies, University of Bradford, Bradford, UK; 3 Department of Health Sciences, College of Health, Medicine and Life Sciences, Brunel University London, UK; 4 Sydney School of Health Sciences, Faculty of Medicine and Health, University of Sydney, Australia; 5 Care Policy and Evaluation Centre (CPEC), The London School of Economics and Political Science, UK; 6 School of Psychology, University of Surrey, UK; 7 NIHR Applied Research Collaboration South-West Peninsula, University of Exeter, Exeter, UK

**Keywords:** Dementia, Caregiving, Longitudinal, Narrative analysis, Identity, Transitions

## Abstract

Longitudinal studies can provide insights into how family members negotiate the caring role and carer identity over time. Within longitudinal, qualitative interviews on ‘living well’ with dementia from the IDEAL cohort study, the aim of the analyses was to identify the shifting, embedded narratives of family members of people with dementia as they negotiated the caring role and carer identity over time. 20 semi-structured, qualitative interviews were conducted with family members of people with dementia, 14 repeated one year later, and analysed using cross-sectional and longitudinal thematic and structural narrative analyses. Longitudinal, interrelated themes, including the care needs and decline of the person with dementia, relationship change, and variable service support, framed the narrative types of family members. Six shifting narratives, apparent as dominant and secondary narrative types, characterised negotiating the caring role over time: absent/normalising, active role adoption/carer identity, resistance, acceptance and resignation, hypervigilance/submergence and role entrapment, and foreshadowed future. The presence or absence of a carer identity was also evident from interviewees’ accounts, although, even where family members were overburdened by the caring role, they did not necessarily express a carer identity. Rather than considering transition into a carer identity, hearing different narratives within the caring role is important to understand how family members experience caring, whether they see themselves as ‘carers’, and when and how they need support. Timely and continued post-diagnostic support, where different caring narratives are recognised, is needed, as well as international initiatives for carer identification.

## Introduction

It is estimated that one in three people will care for someone with dementia during their lifetime (NHS England, 2023a). The International Alliance of Carer Organizations has defined ‘a caregiver, carer or family caregiver [as] an unpaid individual, such as a family member, neighbour, friend or other significant individual, who takes on a caring role to support someone with a diminishing physical ability, a debilitating cognitive condition or a chronic life-limiting illness’ (IACO, 2024). Caring for a family member with dementia can be a demanding role. Higher stress and depressive symptoms are associated with poorer psychological and physical well-being in family members caring for people with dementia (Gilhooly *et al*., 2016; Rippon *et al*., 2020), with higher rates of psychological sequelae than among family members caring for those with other chronic conditions (Schoenmakers, Buntinx and Delepeleire, 2010; Hopkinson *et al*., 2018). Family caregiving in dementia can have positive aspects (Lindeza *et al*., 2020; Quinn and Toms, 2019) including personal growth (Lloyd, Patterson and Muers, 2016; Quinn *et al*., 2024) and finding meaning (Quinn, Clare and Woods, 2010) which are associated with family carer well-being (Quinn and Toms, 2019). However, there may be variation in the caregiving experience across different countries due to differing cross-cultural norms, national policies and formal support systems (Zarzycki *et al*., 2024).

Longitudinal quantitative studies indicate that predictors of increasing caregiver burden include decreased carer well-being, severity of neuropsychiatric symptoms in the person with dementia, duration of caregiving, and the degree of emotional support from other family members (e.g. Ransmayr *et al*., 2018; Lethin *et al*., 2020; Lindt, van Berkel and Mulder, 2020; Malhotra *et al*.,2024). However, the link between family carer quality of life and neuropsychiatric symptoms is not consistent (Farina *et al*., 2017; Clare *et al*., 2019), perhaps because neuropsychiatric and behavioural symptoms are insufficiently specified across studies (Gilhooly *et al*., 2016). Moreover, in the IDEAL cohort study, although there was a ‘Declining’ class trajectory whose depreciation in ‘living well’ was associated with factors including carer stress and depression (Clare *et al*., 2022), two thirds demonstrated relatively stable ‘living well’ scores (quality of life, well-being and satisfaction with life) over 24 months.

Qualitative systematic reviews have highlighted how carers may feel a ‘hostage’ to the caring role (Macdonald *et al*., 2020) but also how spouses accommodate changes in everyday life due to dementia and within the marital relationship (Egilstrod, Ravn and Petersen, 2019; Pozzebon, Douglas and Ames, 2016; Wadham *et al*., 2016). In their review, largely of cross-sectional qualitative studies, Zhu *et al*., (2024) report how spouses may not adopt a ‘carer identity’ because they regard caring as an integral stage or continuation of the relationship. ‘Carer identity’ itself is defined as ‘a cognitive construction that develops when individuals characterize themselves as a caregiver to a family member or friend, normally for those in need of support due to chronic illness or disability’ (Funk, 2019, pg.1). Certainly, Milne and Larkin (2015) argue that ‘as caring is integral to many relationships, the distinction between caring as a normative activity and an activity beyond the normative is problematic’ (pg.7). Differing cultural narratives are reflected in the shift from ‘carer’ to ‘care partner’ as the preferred term in U.S. dementia care and research, particularly for family and friends of individuals requiring minimal support such as those early into the disease trajectory (Gitlin, Jutkowitz and Gaugler, 2020; Jeste, Mausbach and Lee, 2021). In the UK, the term, ‘carer’, has remained in common use (e.g. NHS England, 2023b), despite the call for its abandonment since it fails to reflect the nuances of the caring relationship (Molyneux *et al*., 2011).

Crucially, identifying as a carer is central to accessing support and financial assessments but not all family members adopt this label and therefore are less likely to ask for support (Glasby and Thomas, 2018; McAllum *et al*., 2021). The label ‘carer’ may also be divisive to family relationships (Hughes, Locock and Ziebland, 2013; Molyneaux *et al*., 2012). It may signify a new power differential and shift in responsibility within spousal dyads, therein ‘dichotomising the couple’ (Wadham *et al*., 2016: 471) and threatening couplehood (Hellström, Nolan and Lundh, 2007; Molyneaux *et al*., 2012). Other reasons for *not* identifying as a ‘carer’ may be allied to reasons for taking on the role such as family relationships (Greenwood and Smith, 2019), filial piety (Quinn, Clare and Woods, 2010), and role acculturation, particularly related to gender norms (Eifert *et al*., 2015; McAllum *et al*., 2021); for example, older male spouses not identifying as carers (Milligan and Morbey, 2016). Family members of people with dementia may also link ‘carer identity’ to the intensity of the role – i.e. supporting all aspects of daily living - or with being a paid carer (Zhu *et al*., 2024). Conversely, identifying *as* a ‘carer’ may be linked to cognisance that one’s own needs are now subjugated by those of the person with dementia (O’Connor, 2007; Eifert *et al*., 2015) rather than being able to ‘boundary’ or separate the caring role and pursue one’s own meaningful activities (Cherry *et al*., 2019). Studies of carer online identity construction on carer websites and in blogs have highlighted different carer identities such as ‘prisoner’, where family carers feel trapped by the caring role (Cooper, 2021), and ‘guardian of their relative’s selfhood’, where family carers try to preserve their relative’s personhood, with different carer identities remaining stable over months or even years (Prato, Abley and Adamson, 2022). However, in both these studies, the differing online carer identities adopted were by family members who had already assumed the ‘carer’ label rather than by those who had not.

Evidently, ‘carer identity’ may be more nuanced, fluid and temporally-situated than afforded by carer identity theory (Montogomery and Kosloski, 2009; Montgomery *et al*. 2011; Montgomery and Koloski, 2012) which presumes carer identity develops over time with the demands of the caring role. By contrast, positioning theory has been applied in carer identity research (e.g. Morgan *et al*., 2021), where individuals use discourses to position themselves, presenting ‘rights’ for themselves and ‘duties’ on others (Harré, *et al*., 2009; Knowles *et al*., 2016; O’Connor, 2007). Morgan *et al*. (2021: 1) draw on positioning theory to describe how ‘calling forth the carer identity’ is a co-constructed, discursive practice or ‘carering’ between individuals and policy makers, researchers, and broader cultural narratives. For example, family members may be positioned as ‘carers’ by researchers and healthcare professionals to ensure care provision (Henwood, Larkin and Milne, 2018). Such accounts are suggestive of the tension where ‘living well’ for people with dementia may be at the expense of that of family members, where the more negative accounts of family caregiving are underplayed within the ‘living well’ discourse (Tolhurst *et al*, 2019).

Further longitudinal, qualitative research is needed to add to understanding of carer identity in family members of people with dementia. This is particularly important, given the shift in emphasis on carer self-identification to pro-active identification of carers in order to target support (Carers UK, 2022; Glasby and Thomas, 2018; House of Commons Health and Social Care Committee, 2021). Such research is to understand how family members in a caring role for people with dementia negotiate the role over time, its potential impact on their well-being, and what influences if or how they adopt a carer identity. Using longitudinal, qualitative interviews on ‘living well’ with dementia from the IDEAL cohort study, we aimed to identify the shifting, embedded narratives of family members of people with dementia as they negotiated the caring role and carer identity over time. Specifically: what impacts ‘living well’ and psychological coping over time for family members of people with dementia? what narratives do family members use to frame the caring role, and do these change over time? and is a carer identity evident at each timepoint?

## Methodological Approach

### Study Design and Ethics

This longitudinal, qualitative study on ‘living well’ with dementia was conducted with family members as part of a larger cohort study, IDEAL (Improving The Experience of Dementia and Enhancing Active Life). IDEAL was approved by Wales Research Ethics Committee 5 (reference 13/WA/0405) and IDEAL-2 by Wales Research Ethics Committee 5 (reference 18/WS/0111) and Scotland A Research Ethics Committee (reference 18/SS/0037). IDEAL and IDEAL-2 are registered with the UK Clinical Research Network (UKCRN), numbers 16593 and 37955. Details of both the quantitative and qualitative IDEAL components are available in the published protocol (Clare *et al*., 2014).

### Sampling and Recruitment

For the quantitative component of the IDEAL cohort study, people with dementia living in the community and their family member study partners were recruited by clinical researchers via UK National Health Service Research Networks across 29 sites in England, Scotland and Wales from 2014-2016. Recruitment was based on a clinical diagnosis of dementia in the mild-to-moderate stages, in accordance with an MMSE (Mini-Mental State Examination) score of 15 or above (Folstein, Folstein and McHugh (1975). Where the person with dementia was willing to take part, with their permission, a key family member was approached to participate.

For the qualitative component of IDEAL, a diverse sample of participants was recruited from the quantitative cohort using maximum variation sampling (Patton, 2014), based on clinical and sociodemographic characteristics of the person with dementia including: a diagnosis of Alzheimer’s, vascular or mixed dementia at age 65 or over, gender, and socio-economic background based on income and previous occupations. (Findings from the qualitative interviews with people with dementia are reported in Stapley *et al*., in press]. The family members of those with dementia interviewed were also invited to take part in and consented to a qualitative interview. No additional sampling strategy was employed for family members.

### Longitudinal Semi-Structured Interviews

Semi-structured interviews were used to cover the same topics across all interviewees, whilst allowing flexibility to follow up on relevant points of interest raised by interviewees themselves.

Interview topics across both interviews included: coping and adapting, family and relationships, wider networks, services and support, change and ‘living well’. Example questions included: What does ‘living well’ mean to you?, ‘How are things for (insert name) at the moment?’, ‘What kind of changes have you noticed in them over the past year?’ and ‘Has your relationship changed?’ [If so…] in what ways?’ Interviewees’ first interviews were also used to aid discussion during follow up interviews, which focused on any possible changes since the first interview. For example: ‘We talked in our last interview about your relationship with (insert name). Do you feel things have changed between you since we last met? In what way?’ ‘Carer identity’ was not asked about specifically, although its presence or absence was evident from the accounts of family members when they discussed the caring role and their relationship with the person with dementia. Also if interview questions on ‘carer identity’ had been asked, these may have triggered performative accounts, where a ‘carer identity’ may have been enacted for the interviewer or conversely resisted, thus perhaps not reflecting interviewees’ everyday experiences. Interviews were conducted in family members’ own homes by an experienced qualitative researcher with a background in medical sociology. All interviews were audio-recorded then professionally transcribed.

### Longitudinal Thematic and Narrative Analyses

Longitudinal qualitative analyses were based on 14 sets of Time 1 and Time 2 interview transcripts, with an additional 6 interview transcripts available for analysis at Time 1 only, where interviewees had not completed a follow up interview due to participant attrition. Both sets of interviews were summarised in grid format by the analyst, SSt, to aid familiarisation and data management (Braun and Clarke, 2006; Clarke and Braun, 2022) and enable first and second interview content to be contrasted. Thematic analyses were completed cross-sectionally and longitudinally (Derrington, 2018; Thomson and Holland, 2003) using NVivo (2020) to enable inductive insights into group and individual experiences (Tuthill *et al*., 2020).

Transcripts from the 6 participants interviewed at T1 only were included in the cross-sectional, thematic analysis, both to add to the corpus of data and hence facilitate data saturation as well as to ensure that these participants’ contributions to the study were included. Longitudinal descriptive themes from the analyses relevant to ‘living well’ and psychological coping with the caring role were finalised by contrasting T1 and T2 thematic analyses to determine which themes remained dominant longitudinally.

Within-case longitudinal, narrative analyses were also employed to explore family members’ experiences of the caring role, how it was negotiated over time, and whether a ‘carer identity’ was evident from their accounts. We employed holistic-form structural narrative analyses (Lieblich, Tuval-Masiach, and Zilber, 1998; Smith, 2016) which Frank (2012) terms ‘dialogical narrative analysis’. This approach enabled us to focus on family members’ embedded or latent narrative types on negotiating the caring role within their discussions of ‘living well’, in which themes from the longitudinal, thematic analyses are interwoven; i.e. how family members narrated their caring experiences and if this changed over time. This addresses the criticism of structural narrative analyses being at the intrapersonal level only (Murray, 2021) and moves towards understanding how individual narratives are shaped by interpersonal and sociocultural influences.

Through longitudinal, holistic-form structural narrative analyses, focus was on identifying dominant and secondary narrative types, and whether these shifted over time. A ‘dominant narrative type’ was interpreted as such where an interviewee’s way of speaking about the caring role was evident throughout the interview and dominated particularly relevant sections of talk; e.g. when discussing the relationship with the person with dementia. A ‘secondary narrative type’ was classified as such where this type was also evident in how an interviewee discussed the caring role but was secondary to or less apparent than a dominant narrative type. Varying, even opposing narrative types or genres can co-exist (Frank, 2013), therefore the narrative types derived are not positioned along a continuum.

A narrative typology to characterise negotiating the caring role was developed from the analyses of both datasets. In narrative analyses of the Time 1 and Time 2 interviews respectively, analytic attention was paid to identifying a clear narrative line, apparent throughout each participant’s interview, for example, by observing repetition, evaluative comments, and participants’ own reflections, then building a narrative typology (Smith, 2016) which ensured fit within and distinctiveness across narrative types (Kluge, 2000). The interview transcripts from the 6 participants interviewed at Time 1 only were included in the cross-sectional narrative analysis to add further data to aid differentiation between the different narrative types identified. The dominant and secondary narrative types of individual interviewees were also contrasted between Time 1 and Time 2 to determine if these had shifted over time. In addition, the presence or absence of a ‘carer identity’ was an interpretive judgement determined by interviewees’ accounts of the caring role at each timepoint; for example, if an interviewee self-referred using the term, ‘carer’, or wanted access to respite care. To facilitate quality within longitudinal, thematic analyses (Nowell *et al*., 2017) and narrative research (Andrews, 2021), CP reviewed a sample of the interviews and took part in ongoing discussions with SSt regarding the analyses. The analyses were also discussed with IDEAL team members. Because of the time gap between data collection and analyses due to research team member changes, it was not possible to request participant feedback.

## Findings

20 family members of people with dementia took part in a first qualitative interview in 2017, with 14 taking part in a follow up interview one year later in 2018. First and follow up interviews were conducted, with first interviews lasting from 41 to 113 minutes, and follow up interviews from 19 to 65 minutes. Our twenty interviewees ranged in age from 40 to 88 and were predominantly White British; ten were male and ten female, reflecting sixteen spousal relationships and four filial relationships (see Table 1). Of their relatives, fifteen had Alzheimer’s, two vascular dementia, two mixed (Alzheimer’s and vascular), and one frontotemporal dementia. Interviewees are referred to using pseudonyms.

Longitudinal, interrelated themes from the analyses relevant to ‘living well’ and psychological coping with the caring role reflect interview accounts at both time points and are interwoven within the family members’ narrative types of negotiating the caring role; for example, within a carer narrative, how a family member discussed coping with dementia decline. To retain focus on carer narrative types, these descriptive themes are not detailed separately but comprise: the health problems of family members (e.g. mobility problems), the care needs of the person with dementia related to dementia decline and other physical health problems (e.g. worsening memory, mobility issues, incontinence), personality change in the person with dementia (e.g. mood changes, loss of inhibition), relationship change (e.g. lack of reciprocal conversation, fewer shared activities), social isolation (e.g. lack of local, family support networks due to geographical mobility), and variable service support (e.g. absence of post-diagnostic support, pushing for adequate social care).

Six narrative types or genres related to negotiating the caring role over time were identified within family members’ accounts of ‘living well’ with dementia: absent/normalising, active role adoption/carer identity, resistance, acceptance and resignation, hypervigiliance/submergence and role entrapment, and foreshadowed future (see Table 2). These are reported as dominant and secondary narrative types, where shifts in narrative types across interview timepoints are evident (see Table 2). Each narrative type, with one or more illustrative exemplars, is presented. The presence or absence of a ‘carer identity’ was also interpreted from these accounts; for example, where interviewees explicitly called themselves a ‘carer’ or from discussions such as wanting respite care. A ‘carer identity’ was apparent within the accounts of all filial but not all spousal interviewees. Notably, for several interviewees, the temporal fluidity of the carer identity was apparent, for example, where this identity was contextualised to specific caring tasks or at times actively resisted to protect the relationship with the person with dementia.

There was also an absence of a carer identity even where some interviewees were finding the caring role demanding.

### 1 Absent/normalising

Absent/normalising refers to a narrative type where the impacts of dementia and the caring role are minimal or absent, and where support for the person with dementia is framed by the spousal or filial relationship. Such ‘normalising’ may be because, in the early stages of the illness, dementia was not greatly impacting the person with dementia and therefore they needed little support. Absent/normalising was the dominant narrative type for 6 interviewees at first interview, particularly for male spouses. Our male spousal interviewees emphasised caring within the context of the spousal relationship.

By the time of his first interview, Steven’s wife had had dementia for 3 years, with little perceived decline which he attributed to his wife staying active. Both Steven and his wife avoided the dementia ‘label’, instead preferring the term ‘aphasia’. Steven’s caring role was to remember words for his wife whose frontotemporal dementia had worsened by Steven’s second interview but the impacts of which, perhaps due to this dementia type, were still minimal:

“I know what the problem is, because I live with it everyday. Erm, it’s, it’s not that much of a problem as far as I’m concerned because I’m (pause) good with words. Erm … So I remember words, erm, and can supply them for [my wife]. Part of everyday life” (Steven, T1)

“So it’s getting worse and it will progress no doubt, err, and I’ll be filling in more gaps” (Steven, T2)

Therefore, by “filling in more gaps”, Steven seemed to be employing a cognitive compensatory strategy for his wife which seemed to support their relationship or sense of couplehood. This was also apparent through Steven’s use of the ‘we’ pronoun throughout his interviews to discuss his and his wife’s shared journey with dementia:

“I mean, we, we’ve both lived with this for what, three year plus now. And, yes, it’s getting worse… But not to the extent where it’s being… a sort of… noticeable impact on our lifestyle” (Steven, T1)

Notably, Steven rejected a carer identity because he regarded this as a more intense and ‘stereotypical’ role, so that remembering words for his wife was the only time during which being a ‘carer’ was marginally apparent. He also questioned why family members were positioned as ‘carers’ in surveys about dementia:

“I keep making the point when I do the tick list of four hundred questions or whatever, um, ‘has your role as a carer, carer, this and carer that’ and on one of them I said “I’m not a carer as such, you know, all I do is fill in the odd word that [my wife] cannot remember”. But carer to me is, is a much more intense, um hands on. Um almost a sort of physical looking after, and that’s totally unnecessary. Um so I realise that there’s probably not a word that could replace it, but I am careful to point out occasionally that what they might imply by ‘carer’ is not how I see myself” (Steven, T2)

Another interviewee also emphasised maintaining couplehood with his wife, “we don’t need other people” (Rob, T1), and the shared experience of living with dementia, although, by his second interview, this had become, “we don’t need other people yet” (Rob, T2):

“you know, it’s er, very … very strange to come to terms with, a bit like Alice in Wonderland, I think, you know. But er, you know, we’re coping pretty well with it” (Rob, T1).

Like several interviewees, Rob also missed “proper conversation” with his wife, with this as a marker of relationship change and potential social isolation. Rob also emphasised the normality of their lives, in spite of his wife’s short-term memory problems and mood swings, the latter of which had been particularly severe prior to his wife knowing her diagnosis. Like Steven, Rob himself equated being a ‘carer’ with needing to offer his wife more support:

“In the realm of things, we lead quite a normal everyday life, just a little bit strange. [laughs]… She’s a fully functional person, as she always has been, so … which has taken a lot of pressure off me. If I was having to … er, be more a carer, then that would be a lot different”. (Rob, T1)

By his second interview, his wife had declined, including waking up confused in the night which had been a shock to him: “the change is we’ve just taken one more step down the road” (Rob, T2).

‘Normalising’ was also evident in other ways within interviewees’ accounts such as Peter regarding his wife’s memory problems as “natural progression” due to ageing: “I suppose everybody has the same sort of problems” (Peter, T1). For James, absent/normalising had been his father’s narrative. James had taken on caring for his mother suddenly after his father’s death, with some resentment that he had not been warned about her needs: “the legacy he’s left us” (James, T1). Absent/normalising may also be inadvertently reinforced. Our interviewees mostly reported a lack of follow up after the diagnosis which may minimise opportunities to discuss the care needs of the person with dementia and hence the caring role of family members. Also, as with Rob’s stepson, visiting family members may not immediately notice the difficulties facing the person with dementia and so also overlook the care being provided: “I think he’s noticed now because the longer you spend with her, the more you can see the problems she’s got. Quick chat and then gone, she’s quite normal, no problem” (Rob, T2). In her interview, Jackie spoke about her husband similarly: “I don’t think the children see him as being as much of a problem as I do” (Jackie, T1). Consequently, family members may not fully recognise their own caring role because it is not recognised by others.

### 2 Active role adoption/carer identity

Unlike ‘absent/normalising’ where the impacts of dementia are largely negated, the ‘active role adoption/carer identity’ narrative type describes family members actively taking on the caring role and adopting a carer identity where the role may be discussed positively. This was a single or shared dominant narrative type for 2 interviewees at first interview, both daughters of mothers with dementia, and also a secondary narrative type one other, highlighting how availability of the carer identity may not always be constant but rather temporal and situation-specific.

This was a secondary narrative type for Harriet who discussed now running the carers’ group while their relatives attended memory group sessions:

“it’s rather nice. They have set aside a room for the carers, and we can chat. And now that we’ve been together for over a year, we can really chat…and um, sometimes I…I seem to have taken it over somehow” (Harriet, T1)

Similarly, Esme, whose narrative was dominated by discussion of her caring role, discussed going regularly to a memory group with her mother. Esme also described herself as a carer, “on a Tuesday I’m the carer, so I do all her meds and what have you” (Esme, T1), whilst also actively engaging with her mother’s paid carers during the rest of the week: “I leave them little messages and stuff like that” (Esme, T1). However, by her second interview, Esme had become overburdened by the caring role because her own chronic health condition had worsened.

Within Joan’s account, two dominant narrative types were evident, both active role adoption/carer identity and absent/normalising, where a carer identity was evident within the former only. Although having “freaked out” for the first 3 months after her mother’s dementia diagnosis, Joan had now accepted her mother’s diagnosis, drawing on a carer identity including advocating for better carer support and researching dementia herself: “I’m like Miss Marple, I like to find out” (Joan, T1). Throughout her interview, Joan also positioned herself as a good carer compared with others who she felt regarded Alzheimer’s as “some sort of curse”, and in both interviews, emphasised the importance of adjusting to the caring role:

“You’ve got to adjust your attitude completely. Completely. And I see a lot of people who have got, you know, got relatives with this and they’re almost like operating as normal. You can’t. You’ve got to really just get a total change in your attitudes to how you deal with the eccentricities of it and their repetition which can drive you bananas! And you just have to kind of… and try and distract them if they do get distressed. It’s very much, I mean I’ve been doing it for 10 years now. I feel like an old hand”. (Joan, T1)

“You know, it’s all the attitude, it’s all about the attitude”. (Joan, T2)

However, Joan also switched to her other dominant narrative of ‘normalising’ her caring role within the context of the mother-daughter relationship, including reiterating their similar sense of humour and shared activities such as going to the theatre. Joan saw taking on the caring role as “an honour”, wanting to do it and regarding herself as the most patient of her siblings to do so, in spite of a difficult relationship with her mother in the past. For Joan, caregiving for her mother, within their renewed mother-daughter relationship, was a positive experience. Therefore, in Joan’s account, both a carer identity and distancing herself from it due to the filial relationship co-existed, again highlighting the fluidity of the carer identity; although, with her mother’s needs intensifying by the second interview, such distancing was less apparent, with Joan’s carer identity beginning to dominate.

### 3 Resistance

By contrast with actively adopting the caring role and largely as a secondary narrative type, ‘resistance’ reflects how the caring role is compartmentalised in order to protect the spousal or filial relationship or to protect the family member’s own interests and well-being. Resistance was a dominant narrative type at first interview for one interviewee but was identified predominantly as a secondary narrative type. For Esme, with resistance as a secondary narrative, maintaining the mother-daughter relationship was important to her such as going on excursions together, even though Esme’s mother did not always remember what they had done. When asked specifically what ‘living well’ meant to her, preserving their relationship was central: “well, I think the main thing is, it’s trying for us to actually keep the sort of relationship we’ve got” (Esme, T2). For Anne, a former nurse, the caring role had become easier by her second interview, in spite of her husband’s deteriorating mobility, because she was able to adopt a nursing role and therein protect their relationship:

“I think erm, in a funny sort of way sometimes it’s easier, erm I’m a retired nurse… so, I do try and go into nursing mode rather than wife mode. Erm, I don’t have any problems with all these things that can go wrong and in some ways it’s, it’s more settled. Erm, it’s awful, isn’t it? As it gets worse it gets more settled. But I can, I can understand and handle that better in a way, yeah”. (Anne, T2)

For Susan, resistance manifested in trying to protect her needs and self-identity. Her husband’s dependence on her had become difficult, “it’s the clinginess that gets to me” (Susan, T1), where pursuing her own separate interests was important for her well-being: “I think because he doesn’t like to let go of me, I’ve decided I’ve just got to be a bit firmer” (Susan, T1). Similarly, Tom’s wife had become dependent on him and disliked him going out, particularly to his sports club. For Tom, in his 80s, resistance was his dominant narrative type, wanting to keep himself physically and mentally active in older age:

“I am not going to give up my interest because, you know, I don’t want to sit here, day in, day out, and deteriorate”. (Tom, T1)

“I want to try and keep active in the mind as well as my body like, so I play dominoes twice a week at the club which she doesn’t like me going there. But I can’t sit here and be a cabbage. So I want to keep my brain going and meet people etc”. (Tom, T1)

However, by his second interview, Tom had taken on personal care tasks for his wife, her dementia had worsened, and he was now unable to go out until his wife was in bed. Even Tom watching sport on television had been stopped because his wife preferred her own programmes. Therefore, with his wife’s needs increasing and Tom’s caring role intensifying, by his second interview, resistance was apparent as Tom’s secondary, not dominant, narrative type.

### 4 Acceptance and resignation

Instead of resisting the caring role, ‘acceptance and resignation’ refers to accepting the caring role and becoming resigned to its demands, even when such challenges threaten to become overwhelming. This was a dominant narrative type of 4 interviewees at first interview, with most but not all also expressing a carer identity. Faith felt she was coping with her husband’s Alzheimer’s and other health problems, although saying because they both had mobility issues and did not go out much, life now was “just an existence” and “we just miss, I miss all the nice things we used to do” (Faith, T1). This shift in pronoun from ‘we’ to ‘I’ is also suggestive of their changed relationship, with Faith saying she and her husband no longer shared the decision-making and that her husband now remembered more of his marriage with his first wife than with her: “I’m not alone but it’s lonely, if you know what I mean” (Faith, T1). However, Faith seemed resigned to the caring role, with her daughters assuming she was coping, also not wanting the support of paid carers, even after her husband’s hospital stay, because this would encroach on her spousal role:

“So I said, as long as I’m able to do things, I’d want to do it. I wouldn’t really want anybody doing what I saw to be my job” (Faith, T1)

Therefore, Faith discussed the caring role in terms of being a wife rather than drawing on a carer identity, even though she was starting to find the role difficult:

“Unless you’re actually living with it, you don’t, you can’t explain what it’s like really. Um, people say, ‘Oh, look at that poor chap, he’s got Alzheimer’s!’ But they don’t say,’ look at that poor woman, she’s got to look after him! (laughs)” (Faith, T1)

By contrast, Harriet did see herself as her husband’s ‘carer’ but had concerns about taking on this role without follow up support and expertise from his memory clinic:

“…I felt that was a loss because it meant it was another person, with other expertise, keeping an eye on him. Cos sometimes perhaps they think, you know, the carer should know everything. Well, I don’t”. (Harriet, T1).

Harriet did not want her husband going into residential care but did want one- or two-weeks respite to catch up with chores around the home and because she was “so physically and mentally exhausted sometimes” (Harriet, T1). Harriet was also losing out on her own relationship with her grandchildren because her husband could only tolerate them visiting for short periods. Therefore, Harriet saw looking after her husband as a full-time job and was resigned to the role, even though this meant her own needs had become eclipsed in the process:

“Knowingly or unknowingly, he has become the centre of which I am spinning around.

And I’m doing the best… I’m not a nurse, and I’m not a doctor. But I’ve got a lot of common sense”. (Harriet, T1)

With acceptance and resignation, family members may be finding the caring role difficult, but they seem resigned to it and do not ask for or expect support or even know how this can be accessed. With this quieter narrative than the next to be discussed, and so because they seem to be coping, family members with this narrative type may be unheard and overlooked.

### 5 Hypervigilance/submergence and role entrapment

This was the dominant narrative type of 7 interviewees at first interview, characterising dimensions of how the demands of the caring role may become difficult to cope with or even untenable. Hypervigilance refers to the family member being in a constant state of watchful awareness to accommodate the care needs of the person with dementia. Similarly, submergence refers to how they may feel overwhelmed by the caring role and meeting the person with dementia’s needs. Coping with the demands of the caring role is sometimes expressed as role entrapment, where the family member feels trapped or imprisoned by the caring role. Family members may also describe the caring role as distressing which was evident during some of our interviews including where interviewees became visibly upset.

Hypervigilance was experienced by family members including Jackie who discussed “a continual battle” (Jackie, T2) to keep her husband drinking enough to stay hydrated. She also faced “this continual battle to try and keep him stimulated” (Jackie, T2) rather than asleep all day. For Leila, whose husband could not be left home on his own, shopping trips to the supermarket had become challenging because her husband needed help spotting the items they wanted on the shelves: “by the time I’ve had an hour in [the supermarket], I’m really, really at my end, you know. I really, really am. But I can’t leave him here” (Leila, T1). Susan’s stress was compounded by not knowing when she was going to get a break. Having been told she would get respite care every 6 weeks, Susan had not had a break in 4 years which she contrasted with staff working in residential care:

“…if I knew for definite I would get [breaks] I’d be able to cope better. I mean for goodness sake, we’re both 81! It’s not as if I’m a young… And it’s like in the homes, they have a break. But when I’m getting up at night and then looking after him all day, he’s 24/7”. (Susan, T2)

Similarly, Trish felt she supported her husband at the expense of her own needs:

“he’s got more of a social life than I’ve got because I’m the one doing the running around…So if I want to go anywhere, I can’t now” (Trish, T1).

Like Harriet also, Susan felt she had been subjugated to support her husband who was becoming “more of a handful” but was unaware his dementia was affecting her too: “he pushes me in the background really” (Susan, T2). Susan also said that, although they could afford it, she had been unable to access appropriate care for her husband. The day centre her husband was going to was reluctant to have him back because he had been upsetting other attendees and walked out: “so it’s making me a prisoner, isn’t it?” (Susan, T1). By her second interview, Faith reported feeling more socially isolated, depressed and trapped in the caring role, reflecting a carer identity by this timepoint, with her narrative shifting from acceptance to submergence:

“Well, there’s no end, you can’t see an end to it, can you? This is the thing, and you sort of think, well, this is your life, this is how it’s going to be for the rest of your life sort of thing, you know” (Faith, T2)

There was also discussion of shifts in the spousal relationship, with Trish saying she thought her husband did not like her anymore. For Susan, her relationship with her husband had also shifted from a marital relationship to more of a friendship: “I care about him but I don’t love him anymore” (Susan, T2).

In her first interview, Grace’s was the strongest hypervigilance/submergence narrative, with pre-existing relationship tensions magnifying her distress and the difficulties in adjusting to her husband’s dementia. She alternated between saying how stressed she got, “I do lose my rag with him” with repeating, “we’re alright” (Grace, T1). Grace missed the person her husband used to be and who would help her around the house:

“Well, they’re not the same, are they? I mean I am used to that now. Because I’ll say, where is my [names husband]? He used to help me”. (Grace, T1)

Yet, even though Grace seemed overwhelmed by the caring role and the most distressed of those interviewed, she did not draw on a carer identity but rather seemed to reject it, not wanting to be paid Carers Allowance, not wanting her new GP to discuss her husband’s diagnosis, and not regarding her husband as ill and needing care:

“It were strange getting money for [names husband], he’s my husband. So he gets the money that’s supposed to be for him but I didn’t want any so please don’t ask me about it because I ain’t doing it” (Grace, T1)

“I am not looking after him, sick, yet, am I?” (Grace, T1)

Rather Grace seemed to regard her support for her husband as a continuation of the spousal role: “[my son] says he relies on you a lot more… but it’s nothing more than what I’ve probably always done” (Grace, T2). By her second interview, Grace also seemed to have shifted more towards an acceptance and resignation narrative, although this could have been a performative device for the interviewer: “we’re fine, aren’t we? I’m sure you think we are” (Grace, T2). Grace also continued to distance herself from a carer identity. This may have been reinforced during her husband’s annual review, where she was told they did not need support, “like she said, we don’t need carers” (Grace, T2), although not needing paid carers did not mean Grace was not a carer herself.

### 6 Foreshadowed Future

This narrative was apparent as a secondary narrative type across most interviews at both timepoints, referring to family members looking to the future and their potential increased caring role which may have been foreshadowed by their evolving experiences of the caring role. The dementia caregiving trajectory was salient for many, although distanced by others. During both interviews, most but not all were anticipating the future as the person with dementia declined: “I am not even going to think about it. No, I’ll cross that bridge when I come to it” (Ruth, T1). However, others were aware that “there’s nothing easy about it, and it’s only going to get more difficult” (Anne, T1), with Jackie wanting to know how future support could be accessed: “I think I’d probably be happier if I, if I knew specifically where the backup’s going to come as he gets worse” (Jackie, T1).

Having largely employed an absent/normalising narrative in her first interview, by her second, Kate’s dominant narrative had shifted to foreshadowed future. Her mother lived with Kate and her partner, with Kate wanting them to develop a strong bond now before her mother’s dementia progressed: “I want them to have a good strong relationship before things get harder” (Kate, T2). Kate expressed concerns about the future, potentially having to give up work to take on the caring role full-time as well as questioning her ability to cope:

“there’s lots of things I think about and worry about but it’s like… it’s more for down the line that I worry, like” (Kate, T2)

“to be honest with you, I’m scared stiff. I don’t know, I don’t know if I can look after her but I’m going to give it a go, you know” (Kate, T2)

For Joan, seeing the impact of other health problems on her mother, such as dealing with a cataract operation, had given her “a taste of what life’s going to be like”, if her mother became ill with other health problems, “and life’s going to be nightmare” (Joan, T1). Therefore, for some interviewees, thinking about the future meant fearing intensification of the caring role. For those already subsumed by caring and perhaps a carer identity, the dementia trajectory and future caregiving may have been too difficult to contemplate.

## Discussion

This study sought to identify what impacts ‘living well’ and psychological coping over time for family members of people with dementia, the narratives they use to frame the caring role, if these change over time, and if a carer identity was evident at each timepoint. ‘Living well’ themes which remained salient longitudinally were the health problems of family members, the care needs of the person with dementia related to dementia decline and other physical health problems, personality change in the person with dementia, relationship change, social isolation, and variable service support. The longitudinal themes interwoven within the narrative types of caring presented suggest how such domains are important longitudinally and cumulatively, even though longitudinal, quantitative scores on living well remain relatively stable (Clare *et al*., 2022). The 6 dominant or secondary narrative types of family members with dementia reflect a range of discussions of dementia caregiving over time. From this as a minimal role (‘absent/normalising’) to accepting the caring role and becoming resigned to its demands (‘acceptance and resignation’) to caregiving demands dominating the lives of family members (‘hypervigilance/submergence and role entrapment’) to considerations of future caring (‘foreshadowed future’). In addition, family members actively took on being a ‘carer’ (‘active role adoption/carer identity’) or conversely compartmentalised the caring role to protect the spousal or filial relationship or their own interests and well-being (‘resistance’).

As well as identifying shifting dominant and secondary narrative types over time, our findings have demonstrated the fluid nature of carer identity; for example, the co-existence of more than one dominant narrative type such as ‘active role adoption/carer identity’ and ‘absent/normalising’, and the protective function of the ‘resistance’ narrative. The fluidity of the carer identity has been suggested in previous cross-sectional work (Morgan *et al*., 2021; Hughes, Locock and Ziebland, 2013), although our longitudinal narrative findings provide additional support. This temporal fluidity of ‘carer identity’ also negates carer identity theory where transition into the carer identity is regarded as uni-directional, fixed and untransmutable (Montgomery and Kosloski, 2009; Montgomery *et al*., 2011; Montgomery and Kosloski, 2012).

However, our work still supports caregiving transitions outlined in ‘caregiving careers’ (Aneshensel and Pearlin, 1995), where focus is on caring roles and stress appraisal rather than carer identity. Such as with some of our interviewees’ ‘hypervigilance-submergence and role entrapment’ narratives, adopting caring roles can be perceived as stressful but may still be distinct from adopting a carer identity, therein countering assumptions that role engulfment leads to carer identity development (Eifert *et al*., 2015).

Key insights in relation to assuming a carer identity may be gleaned from focus on the ‘absent/normalising’ and ‘hypervigilance/submergence and role entrapment’ narratives specifically. First, the ‘absent/normalising’ narrative adopted by our spousal, particularly male interviewees is commensurate with how a ‘carer identity’ may not dominate because caring is accommodated within or seen as a continuation of the spousal relationship (Egilstrod, Ravn and Petersen, 2019; Zhu *et al*., 2024), as was also evident across other narrative types within the study. A gendered response to caring was apparent in our interviews, where, as in previous research, older male spouses did not identify as carers (Milligan and Morbey, 2016), wanting to protect their relationship from the impacts of dementia (Wadham *et al*., 2016), the intrusion of the dementia ‘label’ and from unwanted support and intervention (Arksey and Glendinning, 2007; Stephan *et al*., 2018). For example, Steven and his wife’s preference for the term, ‘aphasia’, rather than ‘dementia’, and Rob’s reluctance to accept support from others. In addition, our work has highlighted how not adopting a carer identity (e.g. in an ‘absent/normalising’ narrative) was potentially reinforced by social interactions with other family members; e.g. adult children not recognising the difficulties faced by their parents due to dementia. Certainly, Smith, Morton and van Rooyen (2022) have highlighted how other family members of the person with dementia may not understand the challenges encountered by the family member in the caring role. It is also possible that other family members may downplay the impacts of dementia as a coping strategy, to resist caring roles themselves, or to enable continuation of ‘normal’, pre-diagnosis family dynamics. Other research has highlighted how spouses and people with dementia themselves may underplay changes due to dementia, pre-diagnosis (La Esandi *et al*., 2018). However, research into intergenerational family relationships in dementia is largely lacking (Fontaine and Oyebode, 2014; La Fontaine, Larkin and Oyebode, 2024).

The ‘hypervigilance/submergence and role entrapment’ narrative is not unfamiliar, resonating strongly with previous work (e.g. carers as ‘hostage’ to the caring role in Macdonald *et al*. 2020). However, in our study, even where family members were overburdened by the caring role, they did not necessarily manifest a carer identity within their interview accounts, again regarding the role as a continuation of the spousal relationship (e.g. Zhu *et al*., 2024): for example, Grace who was distressed by the caring role but seemed to reject a ‘carer identity’. Because of the relationship between role captivity and depression in dementia caregiving (e.g. Givens *et al*, 2014), this is a concern. Therefore, even where family members were overburdened by caring, they did not necessarily manifest a carer identity within their interview accounts and so may remain ‘hidden’ to service providers (Knowles *et al*., 2016). This suggests a caring paradox where those most in need of help do not necessarily identify as ‘carers’ when doing so would trigger access to support. They may not actively seek formal help, particularly older carers (Greenwood *et al*., 2019) or refuse this when it is offered. This paradox is further complicated in that family members may only recognise themselves as ‘carers’ through their interactions with health professionals or those in peer support groups (McAllum *et al*., 2021), perhaps because they are ‘positioned’ as such by them during these interactions (see Henwood, Larkin and Milne, 2018; Morgan *et al*., 2021). Moreover, Ireland and Hughes (2022) argue that family members adopting a carer identity, such as the ‘active role adoption-carer identity’ in our study, may be socially valorised as ‘heroes’ or ‘angels’, although this risks family members adopting a subjugating approach to the care recipient and having to maintain exacting standards of care. In our study, for example, Esme could not maintain this narrative type because of her own increasing health needs.

However, an absence of continued post-diagnostic follow up was largely reported by our interviewees, thus affording them limited opportunity to discuss their ongoing caring role and how they were coping. In England and Wales, service support may be inequitable across different regions and focus on diagnosis and the succeeding first twelve months (Bamford *et al*., 2021). In The Dementia Strategy for Scotland (Scottish Government, 2023), there is also a commitment to providing support but again for one year, post-diagnosis only, when ongoing support to manage a complex neurodegenerative condition is needed. Optimal, holistic post-diagnostic support also varies globally (Gauthier *et al*., 2022; Morrisby, Joosten and Ciccarelli, 2018) which includes variable access to social care (Stephan *et al*., 2018). In the UK, for example, a carer’s assessment by a social worker is the gateway to accessing respite care; although, in our study, one interviewee was still waiting for respite care four years after requesting it. Overt carer burden such as in the ‘hypervigilance/submergence and role entrapment’ narrative may not be easy to ignore. However, the ‘acceptance and resignation’ narrative type, although seemingly positive and to suggest adaptation to the caring role, may reflect family members accepting the role but by subjugating themselves and their own needs. In this way, ‘living well’ for people with dementia may be at the expense of that of their family members (Tolhurst *et al*, 2019). Therefore, ongoing follow up - beyond the first year, post-diagnosis - and contact with health and social care professionals is essential to initiate self-reflection regarding ‘carer identity’ by family members and to enable psychological and practical support for those who may be overburdened by continued caring.

Certainly, recognition of the caring role is a gradual process. Carers UK (2022) report that 51% of carers took over a year to recognise their caring role, and 36% over three years, hence their 2024 ‘This Counts As Care’ TV campaign which aimed to raise awareness of daily caring activities and prompt reflection on whom might be classed as a ‘carer’ (Carers UK, 2024).

Appropriate communication during the annual review, for example, may also enable family members to recognise their role and discuss their support needs which should be a separate discussion from the person with dementia (NICE, 2020a). This may be particularly important for male spousal carers who, as in our research, are less likely than female spousal carers to use the ‘carer’ term and also engage with peer support (Hughes and O’Sullivan, 2017). Adoption of the term, ‘care partner’, rather than ‘carer’ in such discussions may be valuable, although

becoming an active collaborator or ‘care partner’ within the healthcare team (Bennett *et al*., 2017) risks positioning family members within an ‘active role adoption/carer identity’, where their needs outside the caring role may be unacknowledged and unmet (Holt Clemmensen et al, 2021). Recognising whether a carer’s dominant narrative has changed over time is also imperative in such interactions as this may be indicative of coping: e.g. a shift from an ‘absent/normalising’ narrative to ‘acceptance and resignation’ or ‘hypervigilance/submergence and role entrapment’.

With the impetus for ‘carers’ to be identified rather than self-identifying, including by healthcare professionals and the NHS (Carers UK, 2022; Glasby and Thomas, 2018; House of Commons Report, 2021; NICE, 2021), in the UK, the important role of GP (General Practitioner) surgeries in this process has been emphasised because of their central role in community-based care (Cronin *et al*., 2023) and their long-standing relationship with their patients (NICE, 2020b).

Strategies for carer identification include GP reception staff remaining vigilant to family members booking appointments for the person they care for, clinicians noting who attends an appointment with a patient, or searching registered lists of conditions where the person may require care such as dementia or post-stroke (Spencer and Swinglehurst, 2020). However, effective recordkeeping is imperative. Lawton *et al*., (2024) reported a notable disparity between participants who self-identified as a ‘carer’ (10.1%) compared with those identified on participating GP lists (2.7%). Other examples of good practice include asking all new patients if they identify as carers and using messaging on GP visual display message boards (Carers Trust, 2018). In their scoping review, Cronin *et* al., (2023) have called for development of a universal carer assessment tool. They also highlight key strategies for carer identification including appointing a carer champion or carer lead to maintain a carers register, identifying carers at the point of diagnosis as well as remaining vigilant to signifiers of an undisclosed caring role such as mental health and sleep problems. However, they also note challenges with such approaches, not least the additional demands on their workload, but including where the family member or carer is not registered as a patient with the practice, and that less than one percent of UK family carers are identified via general practice (see also Peters, Rand and Fitzpatrick, 2020).

Carer passport schemes which provide a record to identify unpaid or family carers, including in employment settings, may be another useful approach (Carers UK, 2023). For example, to record use of their one week of unpaid carers leave per year for working carers in England, Wales and Scotland under The Carer’s Leave Act (2023). Publicity campaigns such as in libraries or pharmacies (NICE, 2020a) and on television such as the ‘This Counts As Care’ TV campaign already outlined (Carers UK, 2024) may also have impact. However, such approaches may be regarded as still needing carers to self-identify, and, as with our findings, protecting relationships may result in a guarded response to self-identification by family members. Nonetheless, such messaging could address this reticence overtly, emphasising the benefits of carer self-identification such as access to appropriate support and information, and also target family members of people with dementia specifically because of the potential for high care demands within this group. Notably, Cronin *et al*., (2023) report that most of the included studies in their scoping review were conducted by UK researchers and suggest ‘a significant policy-practice gap internationally’ (pp.13) to carer identification and support which future initiatives must aim to address.

### Strengths and Limitations

Our longitudinal study is one of relatively few which has considered negotiating the caring role and carer identity over time for family members of people with dementia, highlighting the importance of different, sometimes co-occurring, narratives within the caring role and the fluid nature of carer identity. There was little change in adoption of a ‘carer identity’ across interview sets, although one year between interviews may be deemed insufficient for such change to have occurred. Commencing qualitative data collection on recruitment to the cohort study and hence earlier into the caregiving trajectory would have been useful. However, this was not implemented because originally sampling for the qualitative interviews with people with dementia reported elsewhere (Stapley *et al*., in press), and hence for the family member interviews, was to be based on positive or negative change in the quality of life of people with dementia on the quantitative survey; although in fact changes in such scores were minimal. The sample size itself for this study may be considered relatively small, although fourteen longitudinal and an additional six cross-sectional interviews is commensurate with achieving 9-17 interviews for saturation within qualitative research (Hennink and Kaiser, 2022). A larger sample would also not have been suitable for the in depth structural narrative analysis (Riessman, 2008), particularly as this was implemented longitudinally. Inclusion of the six cross-sectional interviews also aided differentiation between the different narrative types identified, although a second interview would have provided additional data on how or if a ‘carer identity’ had been adopted over time.

In addition, data collection was conducted prior to the COVID-19 pandemic, during which increased carer stress and additional carer burden were reported (e.g. Pongan *et al*., 2021; Budnick *et al*., 2021). Therefore, family member discussions of caring and ‘carer identity’ during the pandemic may have been different, although all qualitative research is bounded within a specific temporal and sociocultural context. Hence transferability (Guba and Lincoln, 1994), if applied, is judged accordingly and with reference to the sociodemographic profile of participants in the study. Certainly, focus on one dementia type rather than across dementias may have reflected the severity of the needs of the person with dementia and consequently the experience of caring. The persons with dementia being cared for were also predominantly older, therefore the accounts of carers of people with young-onset dementia in relation to carer identity were absent. Finally, although reflective of the IDEAL cohort from which the sample was drawn, we are also aware that all interviewees were White British, so that further work would be needed with a more ethnically diverse sample of participants. Strict protocols were adhered to for informed consent from people with dementia themselves to participate in the cohort study. They were also happy for their family member to be interviewed about ‘living well’ and the caring role. However, although evidenced by family members’ narrative accounts, the presence or absence of a ‘carer identity’ was an interpretive judgement which could be considered an ethical issue of representation (Agarwal *et al*., 2021), as it was not possible to request participant feedback on how their accounts of caring have been interpreted and represented.

Nonetheless, this study adds to the corpus of longitudinal, qualitative research on how family members in a caring role for people with dementia negotiate the role over time and which aspects of ‘living well’ impacted by caring remain salient longitudinally. Our work has identified six shifting narratives used by family carers to depict the caring role which may provide a useful framework to help identify how family carers are coping with the role, if this changes, and when and how family carers require post-diagnostic support for their needs to be met, both inside and outside the caring role. Importantly, our longitudinal study adds to cross-sectional work on ‘carer identity’, suggesting a caring paradox, where those most in need of support do not necessarily identify as ‘carers’, thus reiterating the importance of robust, pro-active carer identification strategies.

## Conclusion

In conclusion, this study is one of relatively few longitudinal qualitative projects where the experiences of family members of people with dementia negotiating the caring role and carer identity have been considered over time, with carer identity regarded as temporally fluid and contextualised. Living well with dementia and psychological coping was manifest within the 6 longitudinal, shifting narrative types of family members of people with dementia in which presence of a carer identity varied, even where family members were struggling with the demands of the caring role. Although the ‘active role adoption/carer identity’ narrative may be the most socially valorised, others such as ‘hypervigilance/submergence and role entrapment’, also need to be heard, as well as ‘acceptance and resignation’ narratives where family members seem to be coping but perhaps only by subjugating themselves and their own needs. Timely and continued post-diagnostic support is needed in which shifting narratives of caring over time are recognised. Although fraught with complexity, development of pro-active strategies and guidelines internationally for carer identification is also needed.

## Figures and Tables

**Table 1:** Characteristics of the family members and people with dementia

Pseudonym	Sex	Age Age (at T1 interview in 2016)*	Ethnic background	Education	Relationship to person with dementia	Person with dementia - dementia subtype	Time since dementia diagnosis (at T1 interview in 2016)*
1. Esme	Female	49	White British	School leaving certificate at age 16	Daughter	Alzheimer’s Disease	3-5 years
2. Tom	Male	84	White British	School leaving certificate at age 18	Husband	Mixed Alzheimer’s Disease/ Vascular Dementia	3-5 years
3. Steven	Male	71	White British	University	Husband	Frontotempor al Dementia	1-2 years
4. Joan	Female	49	White British	University	Daughter	Alzheimer’s Disease	3-5 years
5. Peter	Male	85	White British	School leaving certificate at age 18	Husband	Alzheimer’s Disease	1-2 years
6. Anne	Female	74	White British	School leaving certificate at age 18	Wife	Vascular Dementia	Not available
7. Grace	Female	72	White British	No qualifications	Wife	Alzheimer’s Disease	5-7 years
8. Susan	Female	79	White British	No qualifications	Wife	Vascular Dementia	5-7 years
9. David	Male	71	White British	School leaving certificate at age 18	Husband	Not available	5-7 years
10. Faith	Female	73	White British	No qualifications	Wife	Alzheimer’s Disease	5-7 years
11. Kate	Female	40	White British	School leaving certificate at age 18	Daughter	Alzheimer’s Disease	Less than 5 years
12. Jackie	Female	69	White British	University	Wife	Alzheimer’s Disease	1-2 years
13. Rob	Male	68	White British	No qualifications	Husband	Alzheimer’s Disease	1-2 years
14. Trish	Female	69	White British	No qualifications	Wife	Mixed Alzheimer’s Disease/ Vascular Dementia	1-2 years
15. Sara**	Female	81	White British	School leaving certificate at age 16	Wife	Alzheimer’s Disease	1-2 years
16. Harriet**	Female	80	White (Other)	School leaving certificate at age 16	Wife	Alzheimer’s Disease	Not available
17. Ruth**	Female	68	White British	No qualifications	Wife	Alzheimer’s Disease	5-7 years
18. Leila**	Female	82	White British	No qualifications	Wife	Alzheimer’s Disease	1-2 years
19. Will**	Male	88	White British	University	Husband	Alzheimer’s Disease	3-5 years
20. James**	Male	54	Not available	College	Son	Alzheimer’s Disease	1-2 years

* ’Age’ and ‘Time since dementia diagnosis’ have been estimated from IDEAL survey data collected in 2014. No sociodemographic or clinical characteristics data was collected at the time of the T1 qualitative interviews.

** Completed T1 interview only.

**Table 2:** Longitudinal dominant/secondary narrative types and carer identity

Interviewee	Relationship to Person with Dementia	T1 Dominant and Secondary Narrative Types	T2 Dominant and Secondary Narrative Types	Carer Identity	Longitudinal Shifts in Dominant Narrative Type
1. Esme	Daughter (not live together)	Dominant: active role adoption/carer identity Secondary: resistance; hypervigilance/submergence and role entrapment	Dominant: hypervigilance/submergence and role entrapment Secondary: active role adoption/carer identity; resistance	T1: Yes T2: Yes	Yes
2. Tom	Husband	Dominant: resistance Secondary: acceptance and resignation	Dominant: acceptance and resignation Secondary: resistance; foreshadowed future	T1: Yes T2: Yes	Yes
3. Steven	Husband	Dominant: absent/normalising Secondary: foreshadowed future	Dominant: absent/normalising Secondary: foreshadowed future	T1: No T2: No	No
4. Joan	Daughter (live together)	Dominant: no one dominant type – active role adoption/carer identity; absent/normalising Secondary: acceptance and resignation; foreshadowed future	Dominant: active role adoption/carer identity Secondary: foreshadowed future	T1: Yes T2: Yes	No
5. Peter	Husband	Dominant: absent/normalising Secondary: acceptance and resignation	Dominant: acceptance and resignation Secondary: foreshadowed future	T1: Yes T2: Yes	Yes
6. Anne	Wife	Dominant: no one dominant type – acceptance and resignation; hypervigilance/submergence and role entrapment Secondary: foreshadowed future	Dominant: no one dominant type – acceptance and resignation; hypervigilance/submergence and role entrapment Secondary: resistance	T1: Yes T2: Yes	No
7. Grace	Wife	Dominant: hypervigilance/submergence and role entrapment Secondary: foreshadowed future	Dominant: hypervigilance/submergence and role entrapment Secondary: resistance; foreshadowed future	T1: No T2: No	No
8. Susan	Wife	Dominant: hypervigilance/submergence and role entrapment Secondary: resistance; foreshadowed future	Dominant: hypervigilance/submergence and role entrapment Secondary: foreshadowed future	T1: Yes T2: Yes	No
9. David	Husband	Dominant: no one dominant type - absent/normalising, and acceptance and resignation Secondary: foreshadowed future	Dominant: no one dominant type - absent/normalising and active role adoption/carer identity Secondary: foreshadowed future	T1: No T2: Yes	Yes
10. Faith	Wife	Dominant: acceptance and resignation Secondary: foreshadowed future	Dominant: hypervigilance-submergence (role entrapment and distress) Secondary: foreshadowed future	T1: No T2: Yes	Yes
11. Kate	Daughter (live together)	Dominant: absent/normalising Secondary: hypervigilance-submergence (role entrapment and distress); foreshadowed future	Dominant: foreshadowed future Secondary: absent/normalising; (shifting towards active role adoption/carer identity)	T1: Yes T2: Yes	Yes
12. Jackie	Wife	Dominant: hypervigilance/submergence and role entrapment Secondary: foreshadowed future	Dominant: hypervigilance/submergence and role entrapment Secondary: resistance; foreshadowed future	T1: Yes T2: Yes	No
13. Rob	Husband	Dominant: absent/normalising Secondary: foreshadowed future	Dominant: absent/normalising (shifting towards acceptance and resignation) Secondary: foreshadowed future	T1: No T2: No	No
7. Grace	Wife	Dominant: hypervigilance/submergence and role entrapment Secondary: foreshadowed future	Dominant: hypervigilance/submergence and role entrapment Secondary: resistance; foreshadowed future	T1: No T2: No	No
8. Susan	Wife	Dominant: hypervigilance/submergence and role entrapment Secondary: resistance; foreshadowed future	Dominant: hypervigilance/submergence and role entrapment Secondary: foreshadowed future	T1: Yes T2: Yes	No
9. David	Husband	Dominant: no one dominant type - absent/normalising, and acceptance and resignation Secondary: foreshadowed future	Dominant: no one dominant type - absent/normalising and active role adoption/carer identity Secondary: foreshadowed future	T1: No T2: Yes	Yes
10. Faith	Wife	Dominant: acceptance and resignation Secondary: foreshadowed future	Dominant: hypervigilance-submergence (role entrapment and distress) Secondary: foreshadowed future	T1: No T2: Yes	Yes
11. Kate	Daughter (live together)	Dominant: absent/normalising Secondary: hypervigilance-submergence (role entrapment and distress); foreshadowed future	Dominant: foreshadowed future Secondary: absent/normalising; (shifting towards active role adoption/carer identity)	T1: Yes T2: Yes	Yes
12. Jackie	Wife	Dominant: hypervigilance/submergence and role entrapment Secondary: foreshadowed future	Dominant: hypervigilance/submergence and role entrapment Secondary: resistance; foreshadowed future	T1: Yes T2: Yes	No
13. Rob	Husband	Dominant: absent/normalising Secondary: foreshadowed future	Dominant: absent/normalising (shifting towards acceptance and resignation) Secondary: foreshadowed future	T1: No T2: No	No
14. Trish	Wife	Dominant: hypervigilance/submergence and role entrapment Secondary: foreshadowed future	Dominant: hypervigilance/submergence and role entrapment Secondary: resistance; foreshadowed future	T1: Yes T2: Yes	No
15. Sara	Wife	Dominant: hypervigilance/submergence and role entrapment Secondary: resistance; foreshadowed future	Joint follow-up interview with person with dementia*	T1: Yes T2:n/a	n/a
16. Harriet	Wife	Dominant: acceptance and resignation Secondary: active role adoption/carer identity; resistance	Joint follow-up interview with person with dementia*	T1: Yes T2:n/a	n/a
17. Ruth	Wife	Dominant: absent/normalising Secondary: acceptance and resignation; foreshadowed future	Joint follow-up interview with person with dementia*	T1: No T2:n/a	n/a
18. Leila	Wife	Dominant: hypervigilance/submergence and role entrapment Secondary: absent/normalising; foreshadowed future	No follow-up interview	T1: Yes T2:n/a	n/a
19. Will	Husband	Dominant: absent/normalising Secondary: foreshadowed future	No follow-up interview	T1: No T2:n/a	n/a
20. James	Son (not live together)	Dominant: hypervigilance/submergence (role entrapment and distress) Secondary: active role adoption/carer identity; foreshadowed future	No follow-up interview	T1: Yes T2:n/a	n/a

* The caring role was not discussed in the joint follow-up interviews.

## Data Availability

‘IDEAL data were deposited with the UK data archive in April 2020. Details of how to access the data can be found here: https://reshare.ukdataservice.ac.uk/854317/‘.
